# Altered Differential Control of Sympathetic Outflow Following Sedentary Conditions: Role of Subregional Neuroplasticity in the RVLM

**DOI:** 10.3389/fphys.2016.00290

**Published:** 2016-07-19

**Authors:** Madhan Subramanian, Patrick J. Mueller

**Affiliations:** Department of Physiology, Wayne State University School of MedicineDetroit, MI, USA

**Keywords:** physical inactivity, sympathetic nerve activity, brainstem

## Abstract

Despite the classically held belief of an “all-or-none” activation of the sympathetic nervous system, differential responses in sympathetic nerve activity (SNA) can occur acutely at varying magnitudes and in opposing directions. Sympathetic nerves also appear to contribute differentially to various disease states including hypertension and heart failure. Previously we have reported that sedentary conditions enhanced responses of splanchnic SNA (SSNA) but not lumbar SNA (LSNA) to activation of the rostral ventrolateral medulla (RVLM) in rats. Bulbospinal RVLM neurons from sedentary rats also exhibit increased dendritic branching in rostral regions of the RVLM. We hypothesized that regionally specific structural neuroplasticity would manifest as enhanced SSNA but not LSNA following activation of the rostral RVLM. To test this hypothesis, groups of physically active (10–12 weeks on running wheels) or sedentary, male Sprague-Dawley rats were instrumented to record mean arterial pressure, LSNA and SSNA under Inactin anesthesia and during microinjections of glutamate (30 nl, 10 mM) into multiple sites within the RVLM. Sedentary conditions enhanced SSNA but not LSNA responses and SSNA responses were enhanced at more central and rostral sites. Results suggest that enhanced SSNA responses in rostral RVLM coincide with enhanced dendritic branching in rostral RVLM observed previously. Identifying structural and functional neuroplasticity in specific populations of RVLM neurons may help identify new treatments for cardiovascular diseases, known to be more prevalent in sedentary individuals.

## Introduction

The sympathetic nervous system plays an important role in the control of arterial blood pressure (Guyenet, 2006). Overactivation of the sympathetic nervous system is associated with several cardiovascular diseases (Zucker et al., 2001; Fisher et al., 2009; Malpas, 2010). Although activation of the sympathetic nervous system is traditionally thought to occur in an all-or-none fashion, selective activation of specific sympathetic nerves has been observed during acute physiological conditions such as changes in blood volume (Victor et al., 1989; Carlsson et al., 1992; Ditting et al., 2006; Ramchandra et al., 2009). Furthermore, differential changes in sympathetic outflow occur under pathological conditions such as obesity, hypertension and heart failure (Eikelis and Esler, 2005; Floras, 2009; Malpas, 2010; May et al., 2010). For example, in the angiotensin II-salt model of hypertension, sympathetic nerve activity (SNA) to different vascular beds can be increased, decreased or unchanged (Osborn and Fink, 2010; Yoshimoto et al., 2010; Foss et al., 2013). Similarly, we have shown in two separate studies that sedentary vs. physically active conditions enhance increases in splanchnic but not lumbar SNA in response to activation of the rostral ventrolateral medulla (RVLM) (Mischel and Mueller, 2011; Mueller and Mischel, 2012). Understanding the specific contribution of different sympathetic nerves to certain disease states is an important step in discovering ways to alleviate the deleterious effects of high SNA (Osborn and Kuroki, 2012).

Studies over the past several decades have reported unique patterns of sympathetic outflow, elicited by activation of different regions in the brain (Dampney and McAllen, 1988; Beluli and Weaver, 1991; Dean et al., 1992; Stocker et al., 2005). Among these, the RVLM plays an important role in the tonic and reflex control of blood pressure and SNA (Guyenet, 2006; Pilowsky et al., 2008; Dampney, 2009; Schreihofer and Sved, 2011). Previous studies performed almost exclusively in cats, have demonstrated that activation of specific subregions of the RVLM produces selective activation of specific sympathetic nerves (Lovick and Hilton, 1985; Dampney and McAllen, 1988; Dean et al., 1992; McAllen and May, 1994). Although a subsequent study in rabbits supported these findings (Ootsuka and Terui, 1997), other studies somewhat similarly performed in rats did not provide evidence of differential control of SNA within the RVLM (Hayes and Weaver, 1990; Beluli and Weaver, 1991). More recently, we have reported differential sensitivity of several sympathetic nerves in response to stimulation of the RVLM in rats. Importantly, this study provided individual examples in which SNA could be selectively activated by direct microinjections within the rat RVLM (Mueller et al., 2011). Based on the differences in the sympathetic nerves recorded and other methodological differences between our study and others (Hayes and Weaver, 1990; Beluli and Weaver, 1991), we concluded that further studies were needed to determine the extent and nature of differential control of SNA in the rat RVLM (Mueller et al., 2011).

Physical inactivity is an important risk factor for cardiovascular diseases and has been associated with overactivity of the sympathetic nervous system (Mueller, 2007a, 2010; Martins-Pinge, 2011; Mischel et al., 2015). In addition to previous studies suggesting differential effects of sedentary conditions on regional SNAs (Mischel and Mueller, 2011; Mueller and Mischel, 2012), we have reported that sedentary conditions enhance dendritic branching in bulbospinal RVLM neurons compared to physically active conditions (Mischel et al., 2014). Interestingly, sedentary but not physically active animals demonstrated greater dendritic branching in more rostral compared to caudal subregions of the RVLM. To our knowledge these were the first data to provide direct evidence that neurons located in different subregions of the RVLM are influenced in a structurally specific pattern by different physiological conditions (i.e., physical activity vs. inactivity) (Mischel et al., 2014, 2015). Despite our novel findings of structural neuroplasticity in the RVLM of sedentary vs. physically active rats, *in vivo* functional correlates to the unique pattern of dendritic branching between sedentary and physically active animals are lacking. Thus, the purposes of the present study were to determine whether glutamatergic activation of the rat RVLM produces differential changes in lumbar and splanchnic SNA, and whether sedentary vs. physically active conditions alter regulation of SNA in a pattern consistent with subregional neuroplasticity reported previously (Mischel et al., 2014). Based on our previous structural and functional studies of the RVLM (Mischel and Mueller, 2011; Mueller and Mischel, 2012; Mischel et al., 2014), we hypothesized that inactivity-induced enhancement of dendritic branching in more rostral subregions of the RVLM would correspond to enhanced responses in SSNA but not LSNA in sedentary rats compared to physically active rats.

## Methods

All experimental protocols and surgical procedures were reviewed and approved by the Institutional Animal Care and Use Committee at Wayne State University and conducted in accordance with the Guide for the Care and Use of Laboratory Animals. All animals received *ad libitum* food and water.

Male Sprague-Dawley rats (75–100 g; Harlan, Indianapolis, IN) were housed in individual cages with running wheels (active rats, *n* = 7) for voluntary running activity or without running wheels (sedentary rats, *n* = 7) for sedentary conditions over a period of 11–16 weeks as described previously in our laboratory (Mischel and Mueller, 2011; Mueller and Mischel, 2012). Commercially available running wheels (Techniplast, Eaton, PA) were used in this study and bicycle computers (Sigma Sport, Olney, IL) recorded daily and cumulative running distances. Bicycle computers were calibrated to the running wheel diameter (34 cm) at the beginning of the study and were checked each weekday and one weekend day for proper functioning.

### Surgical procedures

Following physically active or sedentary conditions, rats from both groups were anesthetized acutely with isoflurane (2–3% in 100% O_2_) and catheters were implanted in the femoral artery and vein for the measurement of arterial pressure and administration of drugs, respectively. A tracheostomy was performed and the rats were artificially ventilated with a mixture of isoflurane and oxygen (2–3% in 100% O_2_). Sympathetic nerve recordings were performed as described previously (Mischel and Mueller, 2011; Mueller and Mischel, 2012) with the exception that lumbar and splanchnic nerves were recorded simultaneously in all experiments. Briefly, a midline laparotomy was performed to expose the lumbar nerve. A section of the left lumbar chain was isolated caudal to the renal vein, placed on the electrodes and covered with Kwik-Sil gel (World Precision Instruments, Sarasota, FL). The abdominal incision along with the muscle layer was sutured around the electrodes as they exited the peritoneal cavity. The animal was then rotated to make a flank incision to expose left splanchnic nerve via a retroperitoneal approach. A dissected portion of the postganglionic splanchnic sympathetic nerve was placed on the electrodes and covered with Kwik-Sil gel as described above. After completing the surgeries for electrode implantation, the rats were prepared for RVLM microinjection by placing them in a prone position in a stereotaxic apparatus (David Kopf Instruments, Tujunga, CA). A midline incision was performed at the back of the head and the muscle overlying the skull was dissected. After a partial occipital craniotomy, the underlying atlanto-occipital membrane was incised to expose the brain stem. After completing all surgical procedures, the long acting anesthetic, Inactin (0.025 ml/min, 100 mg/kg iv; Sigma Aldrich, St. Louis, MO) was administered over a period of 30–45 min, and the isoflurane was reduced gradually to zero. After the initial loading dose of Inactin was infused, supplemental doses of Inactin (5 mg, i.v.) were given to prevent withdrawal to toe pinch and to maintain a consistent plane of anesthesia. Animals were artificially ventilated (60–80 breaths/min) with 100% O_2_ for the rest of the experiment. Arterial blood gas levels were determined and maintained within the normal range (Po_2_ > 100 mmHg, Pco_2_ between 35 and 40 mmHg) by adjusting the rate or volume of the ventilator. Body temperature was maintained near 37°C using a heating pad. All the experimental procedures were performed within a Faraday cage to minimize electrical noise.

### Microinjections

Drugs were microinjected similar to methods used in previous studies by our laboratory (Mueller, 2007b; Mischel and Mueller, 2011). Briefly, microinjections were performed using a single-barrel glass micropipette (tip diameter 20–30 μm), mounted on a stereotaxic micromanipulator (Ultra Precise, Kopf; <10 μm resolution in three dimensional planes), and connected to a pressure microinjection system (Toohey Company, Fairfield, NJ). A microscope with a calibrated reticule (<3 nl resolution) was used to determine that 30 nl of 10 mM glutamate was injected into the RVLM at each site by visualizing the meniscus of the pipette barrel of a known diameter. In all studies, the RVLM was initially identified using injection coordinates used in our previous studies (Mischel and Mueller, 2011; Mueller et al., 2011; Mueller and Mischel, 2012). In order to standardize injection coordinates and level the brainstem as described in earlier studies (Kiely and Gordon, 1994; Moffitt et al., 2002), calamus scriptorius was set between −2.4 and −2.6 mm caudal to intraaural zero by adjusting the angle of the animal's head. Once set, calamus scriptorius was used as a reference point and the following coordinates were used to find the RVLM: 1.0 mm rostral and 1.8 mm lateral to calamus scriptorius, and 3.2 mm ventral to the dorsal surface of the medulla. The RVLM was identified initially by a pressor response of ≥10 mmHg to microinjections of glutamate (30 nl, 10 mM or 300 pmol). Our previous studies have demonstrated that repeated injections of glutamate at this volume and concentrations are highly reproducible in the same site when 5 min or more is allowed in between injections (Mueller and Mischel, 2012).

**Figure 1:**
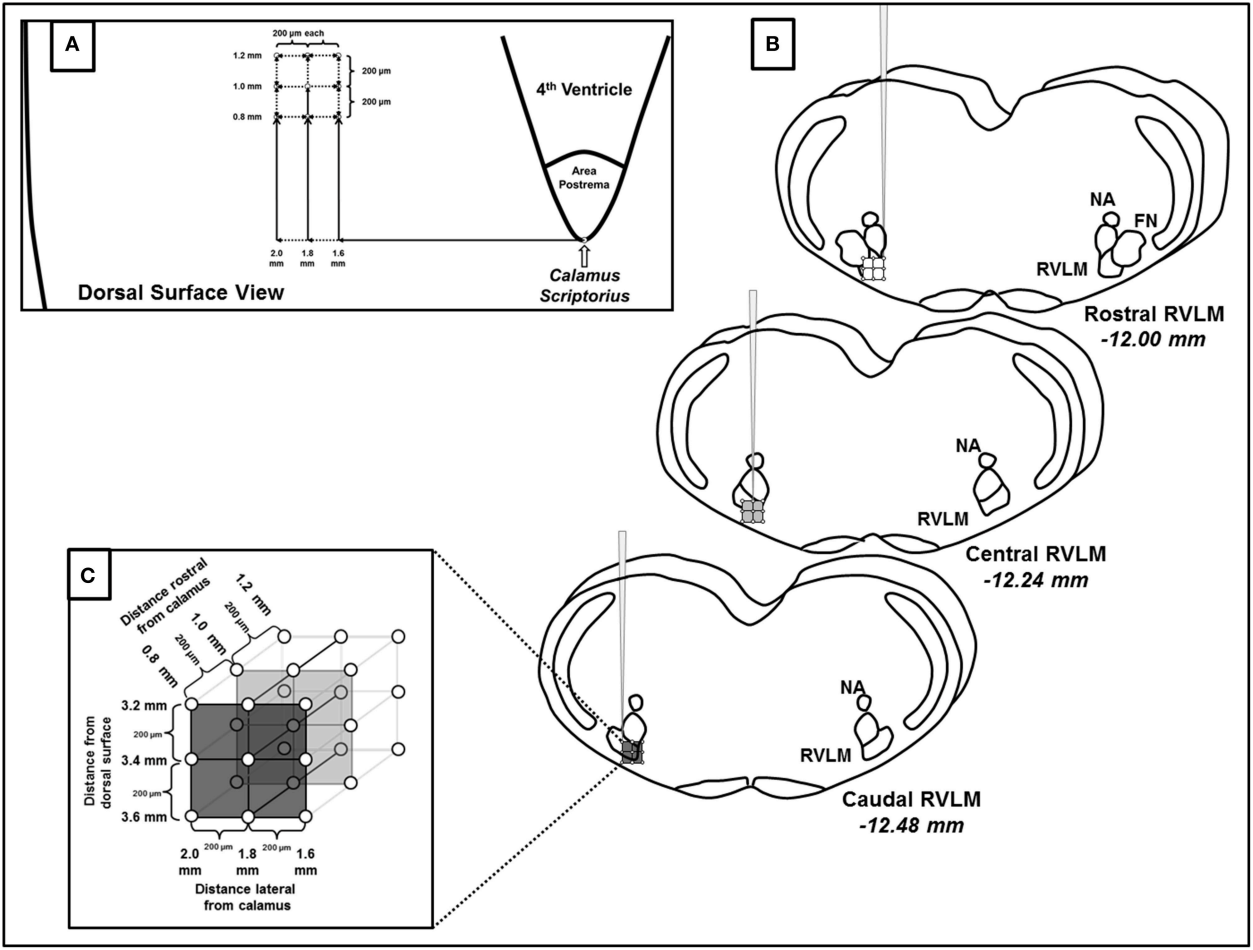
**Schematic representation of microinjections of glutamate made within the rostral ventrolateral medulla (RVLM). (A)** Diagram representing the dorsal surface of the brainstem and the coordinates used to create the microinjection grid pattern. Calamus scriptorius was defined as the region just caudal to the area postrema (AP). **(B)** Three dimensional representation of the rostrocaudal, mediolateral and dorsoventral extent of RVLM microinjections, denoting their position relative to other common brainstem structures observed during histological processing. Diagrams are modified from a standard rat atlas (Paxinos and Watson, 2007) and measurements represent distances from Bregma associated at each level of the RVLM. **(C)** Magnified view of the three dimensional injection grid created by microinjections at the coordinates listed in **(A,B)**. As indicated each injection site was separated by 200 μm. (NA, nucleus ambiguus; FN, facial nucleus).

Following functional identification of the RVLM, we performed a series of microinjections of glutamate of the same volume and concentration (30 nl, 10 mM) in a grid-like fashion (see Figure 1). Specifically, within each pipette track, injections were performed at 200 μm intervals at depths of −3.2, −3.4, and −3.6 mm from the dorsal surface of the brain. The pipette was then withdrawn and moved 200 μm rostrocaudally and/or 200 μm mediolaterally to perform the subsequent injection. The three injections at the same dorsoventral depths were then repeated. This pattern was repeated and injections performed in the rostrocaudal and mediolateral planes were given in a random order with each injection site separated by 200 μm as shown in Figure 1. A minimum of 5 min of recovery time was allowed between injections based on our previous study demonstrating reproducible responses to this same dose and volume of glutamate (30 nl, 10 mM), including repeated injections in the same injection site (Mueller and Mischel, 2012). In addition to preexisting evidence of reproducibility in the same site of injection, we based this injection protocol on our previous study in which this exact same volume and concentration of glutamate (30 nl, 10 mM) produced differential activation of SNA when relocating the pipette as little as 200 μm from the initial site of injection (Mueller et al., 2011).

### Histology

In addition to functional identification of the RVLM with glutamate injections, microinjection sites were also localized by microinjections of 2% Chicago Sky blue dye (30 nl) at the end of each experiment. Dye injections at this volume and concentration were used solely to approximate the center of a single injection as in our previous studies (Mischel and Mueller, 2011; Mueller et al., 2011; Mueller and Mischel, 2012). Because of the high probability of obscuring the location of a single dye injection, no attempt was made to perform dye injections at every injection site of the injection grid.

At the end of the experiment, animals were sacrificed by an overdose of a standard euthanasia solution (Fatal plus, 0.2 ml; Vortech, Dearborn, MI). Brains were removed and placed in 4% phosphate buffered formalin solution. After post-fixation, brains were transferred to 20% sucrose overnight and then 30% sucrose for cryoprotection for at least 48 h. The hindbrain was blocked, frozen and cut into 50 μm sections on a cryostat. Alternate sections were mounted on two different sets of gel-coated slides and either stained with neutral red or left unstained in order to determine the location of anatomical structures or to identify the location of the center of the dye injection, respectively. The center of the dye spot was identified using a compound microscope and then represented as a single point on a modified diagram from a standard rat atlas (Paxinos and Watson, 2007). Because of their different chemical properties and because the dye spread was not intended to be used to determine the relative amounts of neuronal activation occurring while glutamate diffused from the pipette tip down its concentration gradient, no attempt was made to equate the spread of the dye injection with the effective spread or area of neuronal activation produced by glutamate.

### Data acquisition and analysis

All experimental data were collected using a computer-based data acquisition system (PowerLab, ADInstruments, Colorado Springs, CO). Sympathetic bursting patterns of both raw LSNA and SSNA were confirmed by the use of an oscilloscope (Hameg Instruments, Columbia, MD) and audio monitor (Grass Medical Instruments, Quincy, MA) as in previous studies (Mischel and Mueller, 2011; Mueller et al., 2011; Mueller and Mischel, 2012). A Grass preamplifier (P511) was used to amplify and filter LSNA and SSNA signals through low pass (3 kHz) filter and high pass (30 Hz) filters. LSNA and SSNA were rectified and integrated with a time constant of 28 ms using Chart software (ADInstruments). Recordings in which ganglionic blockade (hexamethonium, 30 mg/kg and atropine methyl nitrate, 1 mg/kg, iv) eliminated sympathetic bursting patterns were considered to contain postganglionic SNA. Background noise remaining after ganglionic blockade was subtracted from original recordings to determine post-ganglionic SNA. Resting LSNA and SSNA were reported in mV•s as direct outputs from the Chart software. Changes in LSNA and SSNA were calculated and expressed as peak absolute change and peak percent change. After subtraction of noise from each set of values, peak absolute change was calculated by subtracting a 2 s average of the peak response in each nerve from an average of a 10 s baseline period in each nerve taken just prior to each microinjection. Percent change was calculated as the quotient of the peak absolute change and the 10 s baseline period in each nerve prior to each microinjection and then multiplying by 100. As in previous studies, we used absolute changes in nerve activity to compare groups with different levels of baseline nerve activity (Mischel and Mueller, 2011) and both absolute and percent changes in SNA to compare responses across different types of sympathetic nerves (Mueller et al., 2011).

### Contour maps

MAP, LSNA, and SSNA responses evoked by injection of glutamate into the RVLM were used to create contour maps in SigmaPlot (Version 10.0, Systat software, Inc. Chicago, IL). These contour maps were used to *qualitatively* identify regions within the three dimensional space of the RVLM which, on average, appeared to evoke greater or lesser changes in MAP, LSNA, and SSNA within sedentary vs. physically active groups. In addition, the contour maps were used to emphasize conceptually, the spread of each injection and the likely overlap in the functional effects of each injection with those performed in the immediate vicinity.

### Statistical analysis

Baseline hemodynamic variables and body weights were analyzed using Student's *t*-test (SigmaStat Version 3.5, SPSS Inc., Chicago, IL). Glutamate induced changes in MAP, LSNA, and SSNA were analyzed using a two-way analysis of variance (ANOVA). When ANOVA indicated a significant interaction, differences between individual means were assessed by *post-hoc* Holm-Sidak test (SigmaStat). The between subjects factor of sedentary vs. active groups was grouped with *n* = 7 animals in each; whereas, the within subjects factors of location of injections was grouped within each animal. The injections were compared rostrocaudally at three different levels (L1, L2, and L3). At each level, the injections were collapsed at rostral, central and caudal regions during the analysis. In five of seven sedentary animals and in four of seven physically active animals we were able to complete 27 injections (9 injections at each level). Due to the presence of blood vessels on the dorsal surface of the medulla we performed 18 and 21 injections in the two remaining animals in the sedentary group and 15, 15, and 21 injections in the three remaining animals in the physically active group. For all analyses, a probability of *p* < 0.05 was considered statistically significant. Data are expressed as mean ± SEM.

## Results

### Baseline characteristics in sedentary and active rats

Physically active rats ran an average of 401 ± 69 km over the study period prior to acute experimentation. Average daily running distance, speed and duration were 3.86 ± 0.92 km, 47.4 ± 2.8 m/min and 97 ± 20 min, respectively. Table 1 contains baseline characteristics in sedentary vs. physically active rats on the day of experimentation. Body weights measured under Isoflurane anesthesia and baseline mean arterial pressures (MAP) under Inactin anesthesia were not significantly different between groups (Table 1). Resting heart rate (HR) under Inactin anesthesia appeared to be slightly higher in sedentary animals but this difference did not reach statistical significance (*p* = 0.06). Similar to our previous studies performed in separate groups of animals, absolute voltages for resting LSNA were not significantly different between groups (Mueller and Mischel, 2012); however, resting SSNA voltages were significantly higher in the sedentary rats compared to active rats (Mischel and Mueller, 2011).

**Table 1:** **Effects of sedentary vs. physically active conditions**.

**Group**	**Sedentary**	**Active**
*n*	7	7
Body weight, g	418 ± 13	398 ± 12
Resting MAP, mmHg	117 ± 3	113 ± 6
Resting HR, beats/min	323 ± 11	292 ± 11^*p* = 0.06^
Resting LSNA, mV•s	2.27 ± 0.52	1.85 ± 0.23
Resting SSNA, mV•s	1.15 ± 0.12	0.48 ± 0.15^*^

Values are expressed as means ± SE; n, number of animals. MAP, mean arterial pressure; HR, heart rate; SSNA, splanchnic sympathetic nerve activity; LSNA, lumbar sympathetic nerve activity; mV•s, units of rectified, integrated, and amplified SSNA; Post mortem electrical noise has been subtracted out of resting SSNA and LSNA values. (

* *p < 0.05)*.

### Microinjection experiments

**Figure 2:**
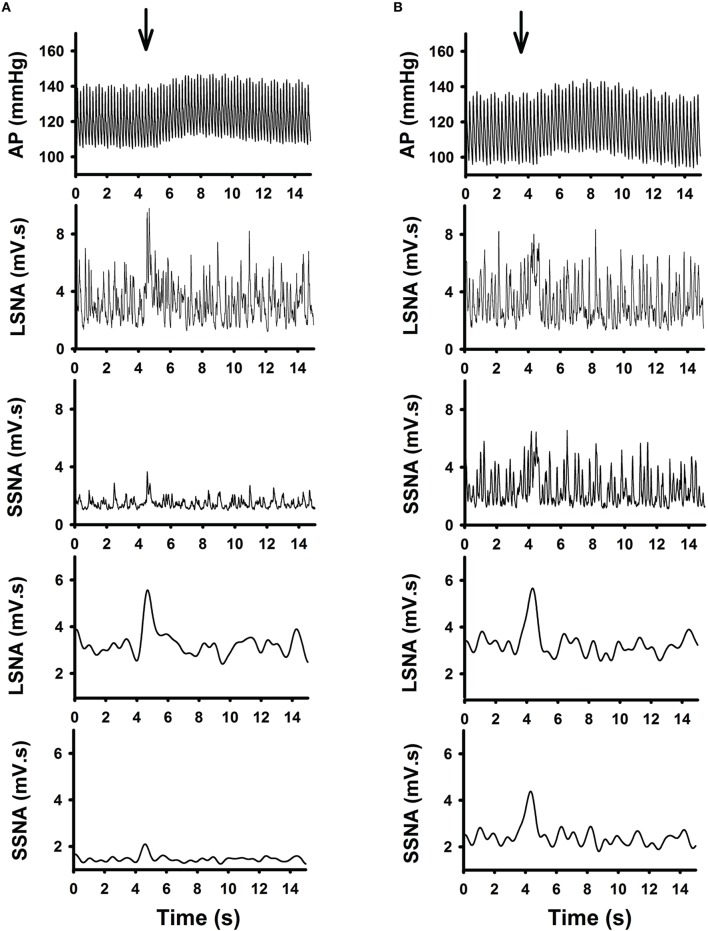
**Representative raw tracing demonstrating the effects of glutamate (10 mM, 30 nl) microinjection in the RVLM from one active (A) and one sedentary (B) rat on arterial pressure (AP), absolute and average changes in lumbar sympathetic nerve activity (LSNA) and splanchnic sympathetic nerve activity (SSNA) were shown**. Glutamate microinjection produced increases in AP, LSNA and SSNA in both groups. Despite similar responses in LSNA between the two rats, the SSNA responses were enhanced in the sedentary compared to the active rat.

Examples of individual microinjections performed in one active and one sedentary rat are shown in Figures 2A,B respectively. Glutamate microinjection produced increases in MAP, LSNA, and SSNA in both groups. Increases in LSNA produced by glutamate were similar between the active and sedentary rat (compare Figures 2A,B, fourth panels); whereas, the SSNA response was much larger in the sedentary compared to active rat (compare Figures 2A,B, fifth panels).

**Figure 3:**
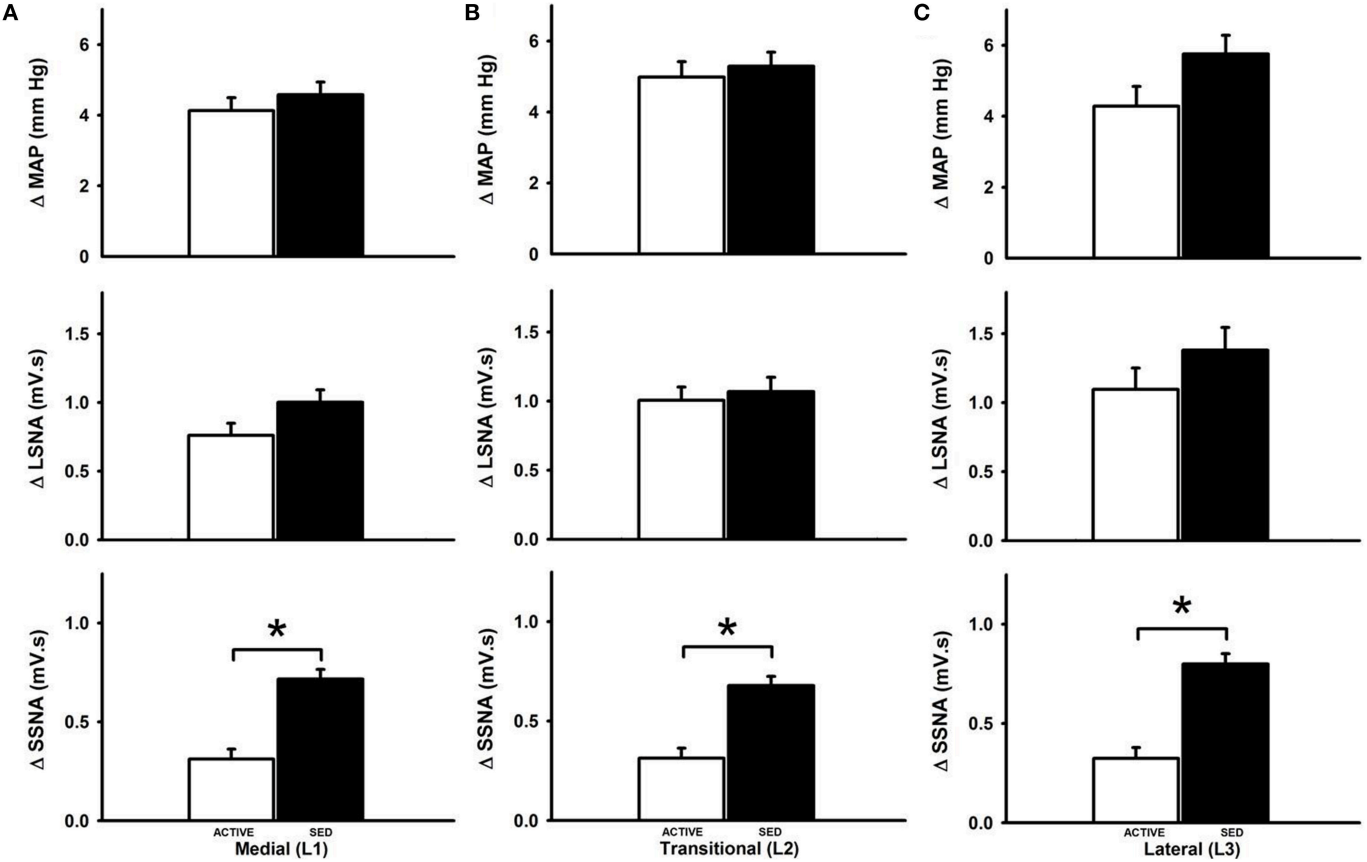
**Average changes in MAP, LSNA and SSNA responses to microinjection of glutamate within the RVLM between sedentary and active rats at medial (L1), transitional (L2) and lateral (L3)**. Active rats (open bars) has similar changes in MAP and LSNA compared to sedentary rats (filled bars) at all three mediolateral levels **(A–C)**. SSNA responses in sedentary rats were significantly greater compared to active rats in all the three levels (^*^p < 0.05).

Figure 3 represents averaged responses in sedentary and physically active animals (*n* = 7 animals each). When responses were analyzed as a function of mediolateral location of injection sedentary conditions did not affect glutamate-induced increases in MAP at the medial (Figure 3A), transitional (Figure 3B) or lateral (Figure 3C) sites of injection (*p* < 0.05 for each). Increases in LSNA in responses to glutamate were also not different between groups at any mediolateral location. In contrast, sedentary conditions significantly enhanced the increase in SSNA at the medial (L1) (Figure 3A), transitional (L2) (Figure 3B) or lateral (L3) (Figure 3C) sites of injection (*p* < 0.001, for each).

**Figure 4:**
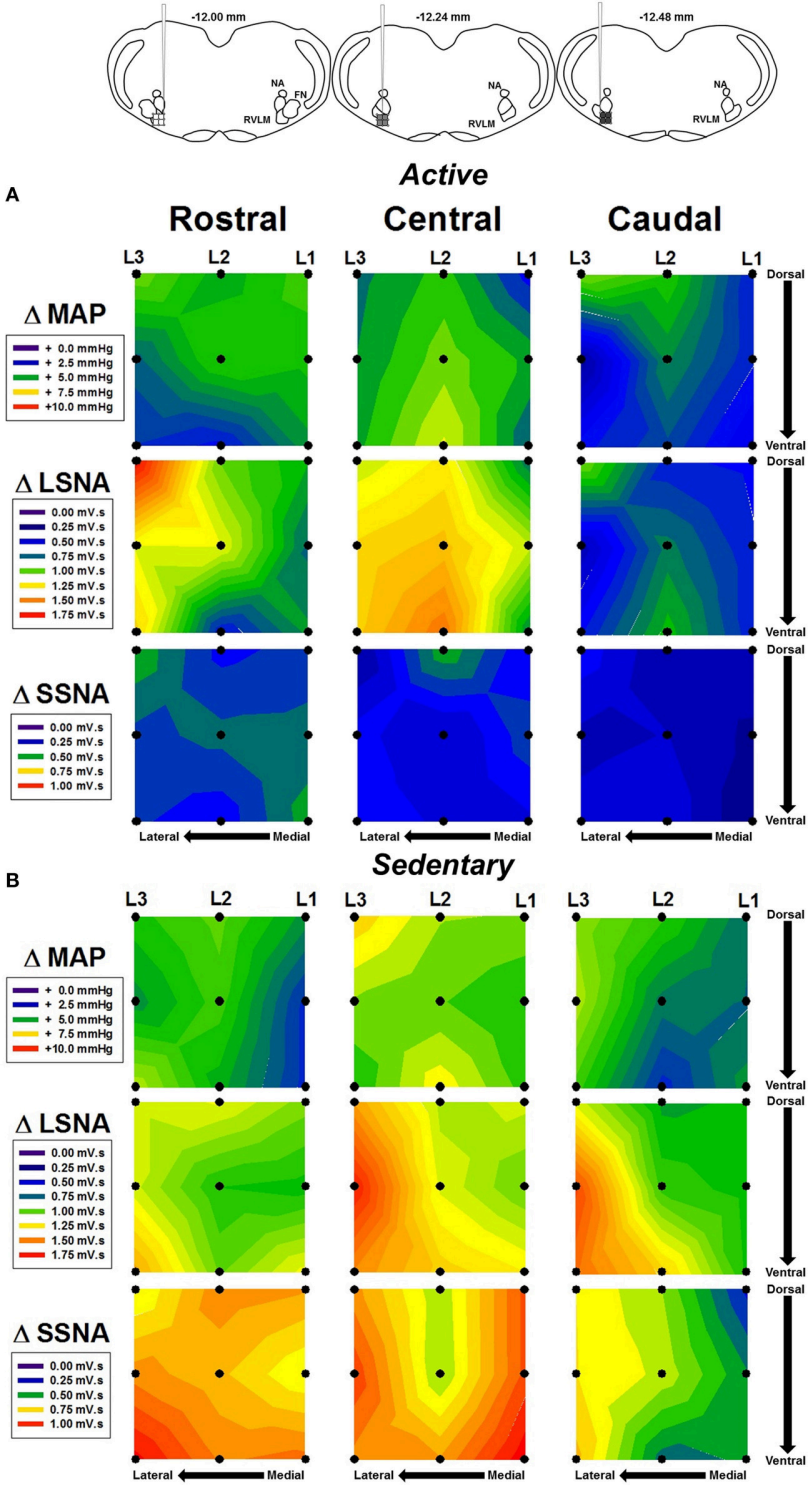
**Average contour maps representing absolute changes in mean arterial pressure (MAP), lumbar sympathetic nerve activity (LSNA) and splanchnic sympathetic nerve activity (SSNA) after microinjection of glutamate (10 mM, 30 nl) into the RVLM**. Diagrams (from Figure 1B) have been replicated above each column to represent the rostrocaudal levels of each set of injections. Each row represents the responses of MAP, LSNA, or SSNA in the rostrocaudal plane for active **(A)** and sedentary **(B)** rats. Filled dots represent location of individual injection sites with each site separated by 200 μm in each plane. Mediolateral levels are shown by L1, L2, and L3 with L1 being most medial and L3 being most lateral.

In order to more fully characterize the relationship between microinjection location and hemodynamic responses to glutamate, contour maps representing the average MAP, LSNA, and SSNA responses in each group were created and are shown in Figure 4. Figure 4A represents the average responses to glutamate in seven physically active rats. Figure 4B represents the average responses to glutamate in seven sedentary rats. Diagrams taken from Figure 1 are reproduced above each rostral, central and caudal level of injection to approximate the rostrocaudal level of injection based on the group averaged results from post mortem histological examination (see below). Within a given variable in each row (e.g., MAP, LSNA, SSNA in horizontal rows), each panel represents microinjections that were performed at a given rostrocaudal level denoted at the top of each column. Within an individual panel, injections performed at the lateral, transitional and medial levels are represented from left to right by L3, L2, and L1 with the highest number representing the most lateral injection. Darker colors (blue and green) represent smaller cumulative responses whereas brighter colors (yellow, orange, red) represent larger cumulative responses (see scales in individual legends for each variable). Qualitatively, and similar to statistical outcomes above, sedentary rats had larger SSNA responses compared to physically active animals (compare third rows in Figure 4A vs. Figure 4B). In addition, areas with the largest SNA responses (red and orange) appeared to differ between SSNA and LSNA responses within sedentary animals such that more rostrally injected areas (left-most SSNA panels) seemed to produce larger responses in SSNA. LSNA responses were more variable between groups such that trends for larger responses only appeared in caudal and lateral areas.

**Figure 5:**
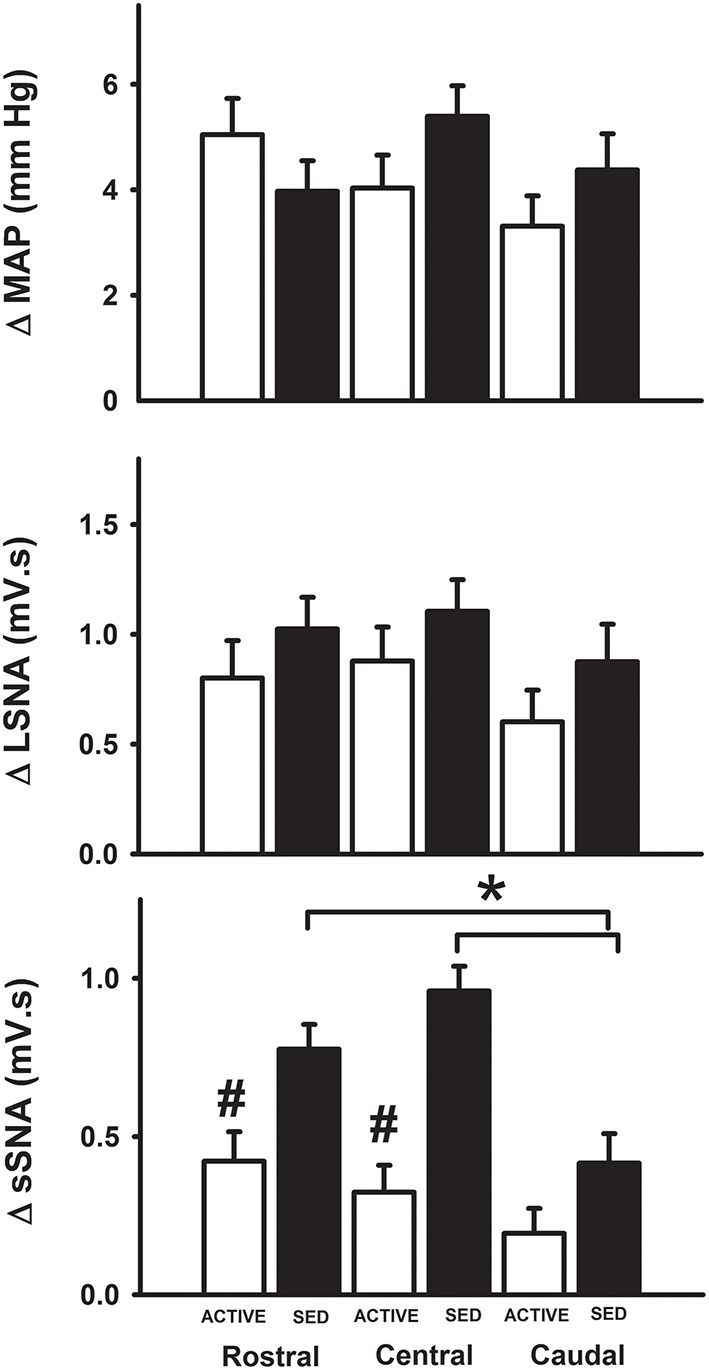
**Average changes in MAP, LSNA, and SSNA in response to glutamate injections based on the rostrocaudal location of injections within the RVLM**. MAP and LSNA responses were not different but SSNA responses were significantly enhanced in the rostral and central regions compared to caudal regions of the RVLM in sedentary animals (^*^p < 0.05; filled bars) and SSNA responses at these levels were also significantly greater than those observed in active animals (#*p* < 0.05; open bars).

Since subsequent analysis of dorsoventral injection location did not reveal differences (data not shown), we analyzed responses based on the rostrocaudal location of the injection site. At the medial sites of injection only, microinjections of glutamate in the rostral and central regions of the RVLM produced significantly greater increases in SSNA in sedentary animals compared to the caudal region in sedentary animals (see black bars, Figure 5). Rostrocaudal differences were not observed for MAP, LSNA and SSNA at the transitional and lateral sites in sedentary or physically active animals (data not shown). In active animals, subregional differences in MAP, LSNA or SSNA responses were not significant at any of the three levels within the RVLM (Figure 5).

**Figure 6:**
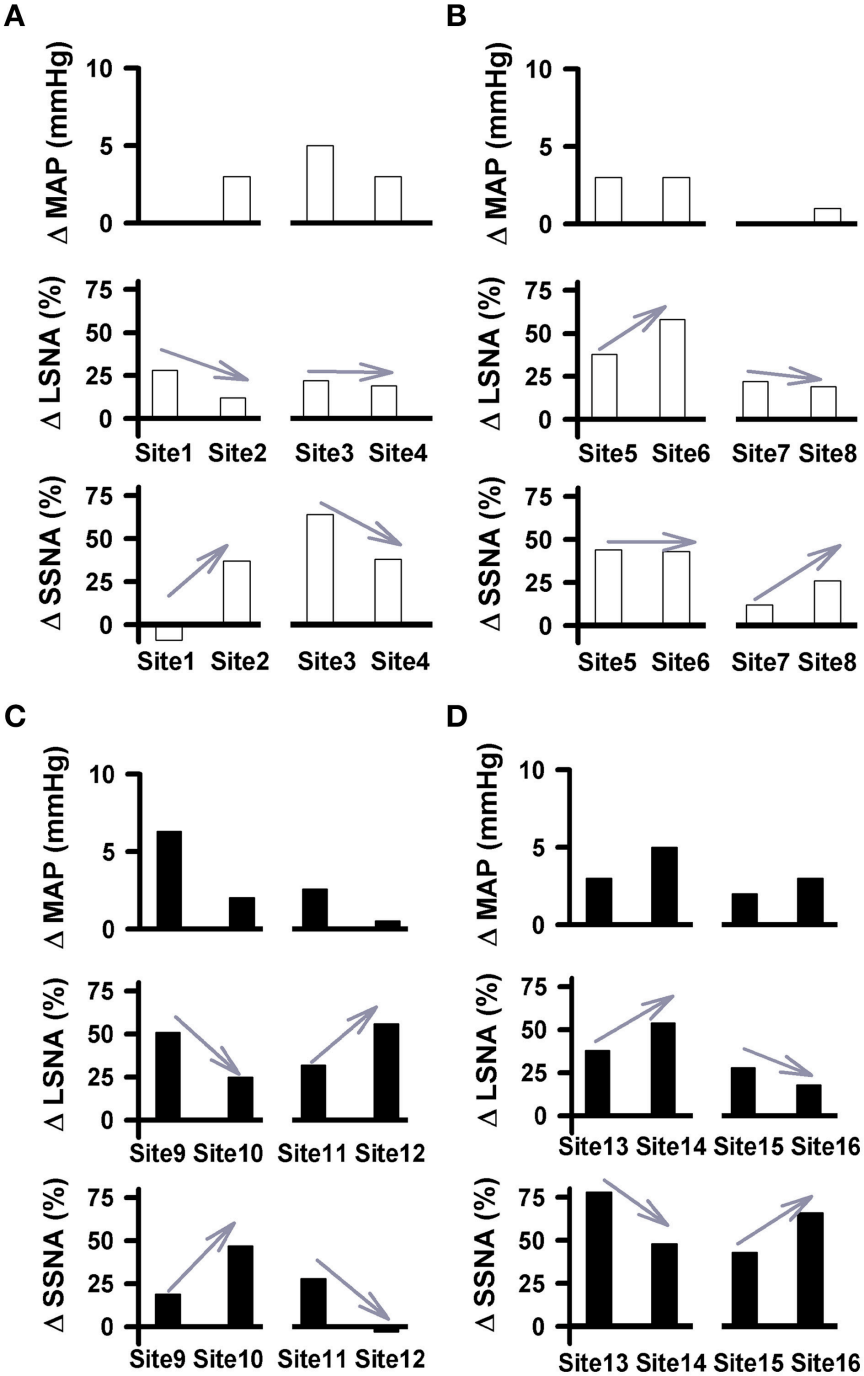
**Pairs of cardiovascular responses to microinjection of glutamate (10 mM, 30 nl) into the RVLM demonstrating differential changes in LSNA and SSNA in physically active (A,B) and sedentary (C,D) rats**. Each Site represents the change in MAP, LSNA, and SSNA to glutamate in a given animal followed by a second injection within 200 μm. Gray arrows demonstrate the direction of the changes in nerve activity from one injection site to another. Paired examples demonstrated instances of directionally opposite changes in SNAs suggesting differential control produced at the level of the RVLM. No change or minimal changes in one nerve and substantial changes in the other suggest that activation of one nerve can occur independent of activation of the other.

Similar to our previous study (Mueller et al., 2011), simultaneous recordings from LSNA and SSNA in the same animal offered us another opportunity to report how individual nerves respond to the same injection of glutamate under identical experimental conditions. Examples of differential changes in LSNA and SSNA as percent change in active and sedentary rats are shown in Figure 6. In each example, injection sites are separated by 200 μm in rostrocaudal, mediolateral or dorsoventral plane. We observed instances where glutamate injections increased LSNA but had little or no effect on MAP or SSNA (i.e., Site 1; Figure 6A). When moving the pipette to another location in the same animal only 200 μm away (Site 2), responses converted to a smaller increase in LSNA yet produced modest increases in SSNA and MAP. In some instances glutamate produced increases in both LSNA and SSNA but when the pipette was moved to a new site, complete opposite effects on each SNA occurred. For example, when glutamate was injected in site 9 (Figure 6C), it increased LSNA, and SSNA to a lesser extent; whereas in site 10, located only 200 μm away, SSNA responses were increased further and increases in LSNA were smaller. In sedentary rats, the magnitude of MAP changes (larger or smaller) as well as trends for changes from one site to the next site was typically associated with trends similar for both LSNA (sites 9, 10, 13, 14) and SSNA (sites 11, 12, 15, 16). On the other hand, in the active rats, glutamate-induced MAP changes and trends for changes from one site to the next appeared to segregate with changes in SSNA only (sites 1–8).

### Histological analysis

**Figure 7:**
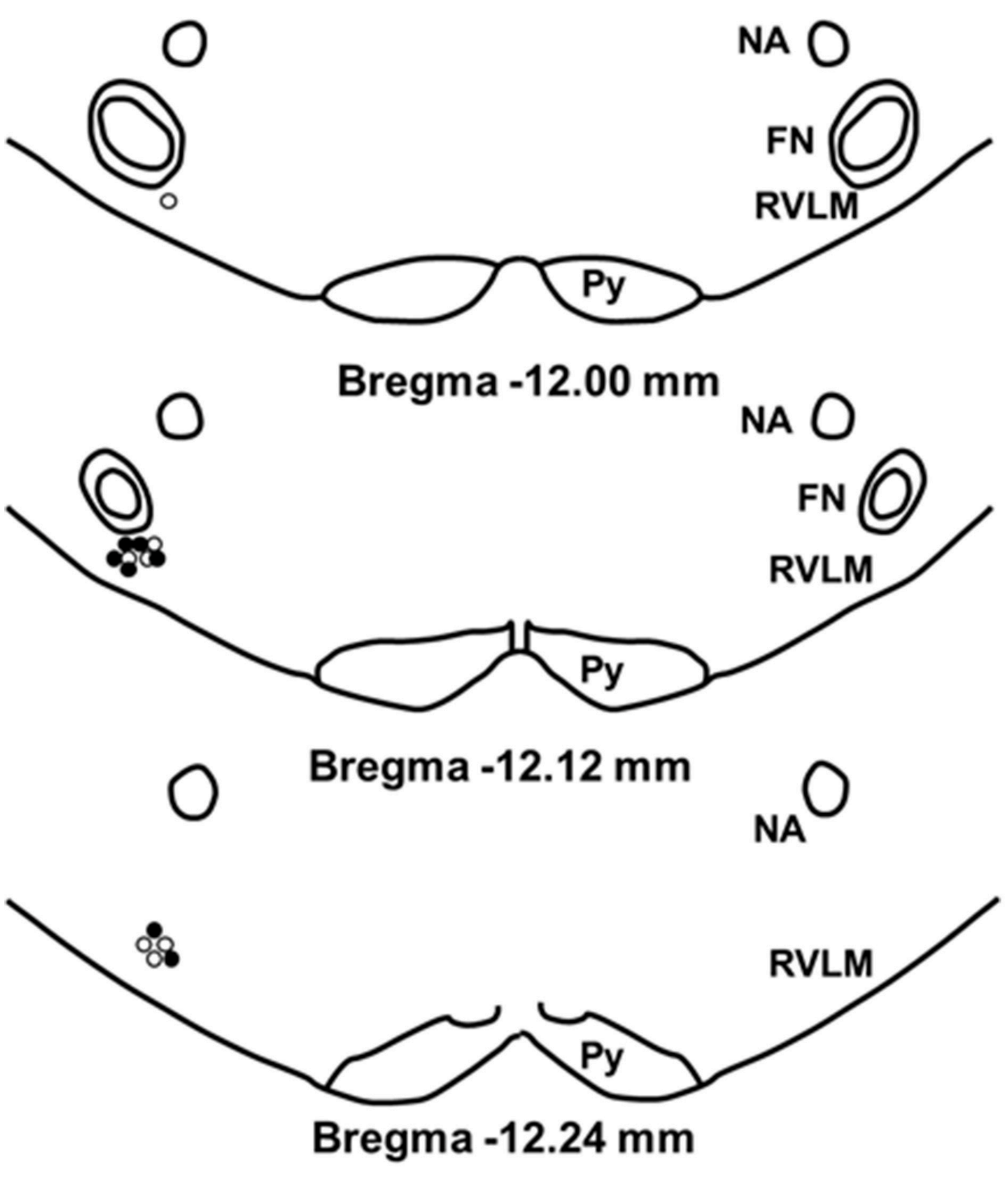
**Schematic representation showing the location of 2% chicago sky blue dye microinjected at the end of each experiment**. Closed circle indicates injection sites from sedentary animals (*n* = 7); open circle indicates sites from active animals (*n* = 7). All injection sites were within 200 μm caudal and 100 μm rostral to the caudal pole of the facial nucleus. (NA, nucleus ambiguus; FN, facial nucleus; Py, pyramidal tract).

To confirm our microinjection sites, we analyzed dye injections (2% Chicago Sky Blue) given at the end of each experiment. Because our injection sites were tightly clustered and the spread of dye does not necessarily duplicate the spread of active concentrations of glutamate, the center of the dye injections was used only to estimate the center of the glutamate injections (Figure 7). Similar to our previous studies, examination of the small open circles (physically active group) and the small filled circles (sedentary group) placed on a modified diagram from a standard rat atlas (Paxinos and Watson, 2007) (Figure 7) revealed no obvious differences in the distribution of injection sites between groups. Also similar to our previous studies, all dye injection sites were determined to have occurred within 200 μm caudal and 100 μm rostral to the caudal pole of the facial nucleus. In previous studies, this region has been described as the cardiovascular area of the RVLM (Guyenet, 2006; Schreihofer and Sved, 2011).

## Discussion

The purposes of the present study were to determine whether glutamatergic activation of the rat RVLM produces differential changes in lumbar and splanchnic SNA, and whether sedentary vs. physically active conditions alter regulation of SNA in a pattern consistent with subregional neuroplasticity reported previously (Mischel et al., 2014). We achieved this purpose by simultaneously recording from two SNAs (SSNA and LSNA), and performing microinjections of glutamate at multiple sites within the classically defined RVLM. Based on our previous structural and functional studies (Mischel and Mueller, 2011; Mueller and Mischel, 2012; Mischel et al., 2014), we hypothesized that sedentary conditions would enhance splanchnic but not lumbar sympathoexcitatory responses to microinjections in more rostral regions of the RVLM. Several novel findings from the present study support our hypothesis and provide important new information related to alterations in neural control of the circulation following sedentary vs. physically active conditions.

First, and consistent with our previous functional and anatomical studies (Mischel and Mueller, 2011; Mueller and Mischel, 2012; Mischel et al., 2014), sedentary conditions enhanced SSNA but not LSNA responses during activation of more rostral and central regions of the RVLM. In active rats, significant region-specific or rostrocaudal changes were not observed, also consistent with our previous anatomical study in which physically active animals demonstrate no change in the pattern of dendritic branching across the rostrocaudal aspect of the RVLM (Mischel et al., 2014). Second, we provide additional evidence that differential control of SNA can be elicited from the rat RVLM. From our initial findings related to adrenal, renal, and lumbar nerve activity (Mueller et al., 2011), we are able to extend these findings to include splanchnic SNA which has been implicated in certain forms of hypertension (Huber and Schreihofer, 2011; Osborn and Kuroki, 2012). Collectively, data from our laboratory demonstrate that sedentary conditions result in region-specific structural neuroplasticity that is associated with region-specific functional neuroplasticity in the RVLM.

The most novel and important finding in the present study is that both sedentary and physically active conditions produced activation patterns of SSNA consistent with the structural neuroplasticity we observed in our previous anatomical study (Mischel et al., 2014). More specifically, our previous study demonstrated that sedentary conditions enhanced dendritic branching in bulbospinal RVLM neurons that project to regions of the spinal cord that control the splanchnic circulation (Mischel et al., 2014). Furthermore, the increased dendritic branching was more evident in rostral regions of the RVLM of sedentary animals (Mischel et al., 2014), consistent with greater SSNA responses to glutamate microinjections in more rostral regions observed in the current study. In contrast, unlike sedentary rats, physically active rats did not exhibit a rostrocaudal variation of dendritic branching in our previous study (Mischel et al., 2014); rather, it was consistent across the entire rostrocaudal extent of the RVLM (Mischel et al., 2014). Correspondingly, we did not observe significant region-specific activation of SSNA in physically active animals in the present study. The consistency of these findings leads us to speculate that structural neuroplasticity in subregions of the RVLM results in significant functional changes that may contribute importantly to the increased propensity for SNA-related cardiovascular diseases in sedentary individuals.

A second important and novel finding in this study builds upon evidence provided by our laboratory that the rat RVLM has the capacity to produce differential activation of SNA. Previously, we reported in non-active rats a differential and selective activation of adrenal, lumbar and renal nerves using microinjections of glutamate of the same size and volume as those used in the current study (30 nl, 10mM) (Mueller et al., 2011). These studies demonstrated a differential control of sympathetic outflows in response to activation of the RVLM whether elicited by direct microinjections or decreases in blood pressure. Similar to the current study, we observed individual instances of selective activation of SNAs after moving the microinjection pipette by as little as 200 μm (Mueller et al., 2011). These studies formed the basis of the mapping strategy used in the present study and allowed us to observe similar instances where changing the injection site as little as 200 μm altered the pattern of SSNA vs. LSNA activation. From these data we speculate that subsets of neurons in the rat RVLM are capable of driving specific pathways that regulate SSNA vs. LSNA, a finding not previously reported by studies in which injections were spaced 500 μm apart and examined splenic and renal sympathetic nerve activities (Hayes and Weaver, 1990; Beluli and Weaver, 1991).

Based in part on our previous anatomical study, one of the primary goals of this study was to map the rostrocaudal boundaries of the RVLM for comparison between sedentary and physically active animals. Therefore, we did not actively seek to identify subregions of the RVLM that were capable of producing selective activation of SSNA or LSNA, although some instances were observed. Certainly, previous studies have demonstrated selective activation of sympathetic nerves via repeated stimulation of specific subregions of the RVLM (McAllen and Dampney, 1990; McAllen and May, 1994; Ootsuka and Terui, 1997). The earliest of these types of experiments were performed almost exclusively in cats (Lovick and Hilton, 1985; Dean et al., 1992; McAllen and May, 1994), with one study reported later in rabbits (Ootsuka and Terui, 1997). In contrast to these findings in larger species, studies by Hayes and Weaver (1990), Beluli and Weaver (1991) reported no evidence of subregional variation within the rat RVLM when recording responses in the renal and splenic sympathetic nerves (Hayes and Weaver, 1990; Beluli and Weaver, 1991). Based on these studies and several neuroanatomical studies (Jansen et al., 1995; Sved et al., 2001; Kerman et al., 2003; Stornetta et al., 2004), a certain “dogma” emerged that the rat RVLM did not have the capacity to produce differential control of SNA. However, as discussed in our more recent report (Mueller et al., 2011), it is likely that the 500 μm spacing of injections performed in the early rat studies may not have had the resolution to identify subregions of the RVLM, particularly because since these studies, the rat RVLM has been more strictly characterized within 500 μm caudal to the caudal pole of the facial nucleus (FN) (Guyenet, 2006; Schreihofer and Sved, 2011). In the present study, the center of our injection sites were always located within 200 μm caudal to the caudal pole of FN as verified histologically. During our experiments the pipette was only moved at 200 μm increments, maximizing our chances of remaining within the confines of the RVLM. Utilizing this approach, we observed instances of directionally opposite responses in sympathetic nerve activities (see Figure 7 for examples). Thus, we contend that these clear-cut examples provide convincing evidence of differential control of SNA by the rat RVLM.

Certainly the technical aspects of our experiments are important to consider. In this study, we injected a specific volume and concentration of glutamate (30 nl, 10 mM), both of which were based on our earlier studies in which we were able to demonstrate instances of differential and selective activation of renal, lumbar, and pre-ASNA (Mueller et al., 2011), as well as obtain reproducible responses with repeated injections (Mueller and Mischel, 2012). Also, similar to our previous study, we were able to reproduce enhanced SSNA responses in sedentary vs. physically active animals (Mischel and Mueller, 2011). Therefore, even though our injection volume of 30 nl was larger than those used in earlier mapping-type studies (5 nl) (McAllen and May, 1994), the primary purpose of our study was not to identify subregions of the RVLM that produced differential SNA responses. Rather we attempted to determine whether region-specific structural changes were consistent with region-specific functional changes between sedentary vs. active rats. In order to more rigorously determine whether specific subregions of the rat RVLM are capable of producing differential and selective activation of different SNAs will require a different experimental approach, perhaps simplified by focusing on only one group of animals.

Because of the similarities in our studies to the earlier mapping type studies, one important comparison is worth noting. Although we did use a larger volume of injection compared to previous studies, we also used much lower concentrations of glutamate (10 vs. 100 mM). Therefore, despite the larger size of our injections, driven by our specific experimental question, the total amount of glutamate per injection used in our study (300 pmol) was 40% lower than that used in the previous mapping studies (500 pmol) (McAllen and May, 1994; McAllen et al., 1995). A fuller discussion and comparison of drug volume, concentration and effective spread is beyond the scope of this work and the reader is referred elsewhere (Mueller et al., 2011; Gaede and Pilowsky, 2012). Nonetheless, based on our work and the work of others, we feel we can conclude confidently that it is highly *unlikely* that glutamate microinjected into subregions of the RVLM: (1) activated the exact same number and populations of neurons and (2) resulted in the same degree of neuronal activation in every neuron. Similarly, although we are confident in our technical abilities to microinject at 200 μm intervals with UltraPrecise™ micromanipulators (<10 μm resolution), we do not claim to have made injections with distinct, non-overlapping spread of glutamate at each injection site. This rationale was the basis for the use of contour maps to qualitatively examine the relationships between injections.

Other technical aspects of our study include our use of both absolute and percentage changes in nerve activity. As explained more fully in our previous studies (Mueller et al., 2011; Mueller and Mischel, 2012), we provided these results in order to effectively compare differences in responses in the same type of SNA across two different groups (i.e., SSNA in sedentary vs. active) and to compare differences in responses in two different nerves within the same group (i.e., SSNA vs. LSNA in sedentary alone or physically active alone), respectively. The use of anesthesia was also required in our experiments to perform multiple injections into different subregions within the RVLM while simultaneously recording LSNA and SSNA. Although RVLM injections have been performed in conscious animals while recording from RSNA (Sakima et al., 2000), it would be highly technically challenging if not nearly impossible to perform our study in conscious animals.

Anatomical evidence supporting differential control of SNA by the RVLM is mixed. For example, several studies quoted by Guyenet (2006) have been interpreted as providing little to no support for an anatomical framework of differential control of SNA by the RVLM (Jansen et al., 1995; Sved et al., 2001; Kerman et al., 2003; Stornetta et al., 2004). In contrast, in a more recent study, injection of different fluorescent tracers into the T4 and T10 segments of the spinal cord of cats resulted in 30% of RVLM neurons being labeled by one or the other tracer vs. about 20% of RVLM neurons RVLM being double-labeled (Gowen et al., 2012). The former result is consistent with the hypothesis that separate populations of neurons may exist within the RVLM to control different SNAs (Mueller et al., 2011). The physiological significance of differential control of SNA is highlighted recently by the proposal that each SNA has different contributions to specific cardiovascular diseases (Osborn and Kuroki, 2012). Alternatively, anatomical studies by Card and colleagues only partially supports the idea of segregation of neurons suggesting that sympathetic output could be derived from a common pathway of neurons within the RVLM, which are altered based on their distinct neural circuits (Card et al., 2011).

## Perspectives and significance

The data from the present study suggest that glutamatergic stimulation within the RVLM of rats results in differential activation of regional sympathetic nerves. Our findings support the idea that a specific subpopulation of neurons within the RVLM of rats has the capacity to drive selective sympathetic outflows. Even though our data did not provide definitive evidence for viscerotopic organization within the RVLM of rats, it is possible that this is related more to the technical challenges of performing microinjections in a smaller species such as the rat. Nonetheless we feel the present study can serve as the basis for future studies examining these possibilities. Our data also reiterate the importance of understanding neuronal plasticity at the level of the RVLM. The region-specific changes in SNA between sedentary and active rats in the RVLM may in turn contribute to the “sympathetic signature” in pathological conditions such as hypertension (Osborn and Fink, 2010).

## Author contributions

MS and PJM were involved in the conception and design of the experiments; MS performed the experiments and analyzed the data; MS and PJM interpreted results of experiments and prepared figures; MS drafted the initial version of the manuscript; MS and PJM edited and revised the manuscript and approved the final version.

### Conflict of interest statement

The authors declare that the research was conducted in the absence of any commercial or financial relationships that could be construed as a potential conflict of interest.
